# Carbonate Doping in TiO_2_ Microsphere: The Key Parameter Influencing Others for Efficient Dye Sensitized Solar Cell

**DOI:** 10.1038/srep23209

**Published:** 2016-03-17

**Authors:** Zaki S. Seddigi, Saleh A. Ahmed, Samim Sardar, Samir Kumar Pal

**Affiliations:** 1 Department of Environmental Health Faculty of Public Health and Health informatics Umm Al-Qura University, 21955 Makkah, Saudi Arabia; 2 Chemistry Department, Faculty of Applied Sciences, Umm Al-Qura University, 21955 Makkah, Saudi Arabia; 3 Department of Chemical, Biological and Macromolecular Sciences, S. N. Bose National Centre for Basic Sciences, Block JD, Sector III, SaltLake, Kolkata 700 098, India

## Abstract

Four key parameters namely light trapping, density of light harvesting centre, photoinduced electron injection and electron transport without self-recombination are universally important across all kinds of solar cells. In the present study, we have considered the parameters in the context of a model Dye Sensitized Solar Cell (DSSC). Our experimental studies reveal that carbonate doping of TiO_2_ mesoporous microspheres (doped MS) makes positive influence to all the above mentioned key parameters responsible for the enhanced solar cell efficiency. A simple method has been employed to synthesize the doped MS for the photoanode of a N719 (ruthenium dye)-based DSSC. A detail electron microscopy has been used to characterize the change in morphology of the MS upon doping. The optical absorption spectrum of the doped MS reveals significant shift of TiO_2_ (compared to that of the MS without doping) towards maximum solar radiance (~500 nm) and the excellent scattering in the entire absorption band of the sensitizing dye (N719). Finally, and most importantly, for the first time we have demonstrated that the solar cells with doped MS offers better efficiency (7.6%) in light harvesting compared to MS without doping (5.2%) and also reveal minimum self recombination of photoelectrons in the redox chain.

The marathon race for the ultimate goal of commercialization of an alternative silicon-free solar cell technology to achieve lower cost-per-watt level with grid parity versus fossil fuel technologies, started with the seminal demonstration of a prototype dye sensitized Solar Cell (DSSC) in last century with 7% efficiency^1^. Since then many efforts are dedicated to enhance the efficiency of the cells by a variety of following ways^2^: (i) selection of strongly absorbing donor-pi-acceptor sensitizing dyes^3^,^4^,^5^(ii) use of redox couples to achieve higher open circuit voltage^6^,^7^,^8^(iii) better thin film (composition and morphology) on the photoanode^9^,^10^,^11^. Apparently first two points look independent of the third one. However, given the amount of dye adsorption, electron injection from the dye to the photoanode and redox coupling, the parameters are found to be key factors for the optimum DSSC efficiency. Successful design of the photoanode again relies on the four key parameter: (a) light trapping via scattering of incident solar radiation (b) very high surface area of the oxide film in the photoanode for dye adsorption (c) ultrafast electron injection from excited dye to the oxide layer (d) efficient transport of charge carriers with minimal recombination loss of electrons^2^.

A mammoth of literature on the various ways of controlling above four parameters for the efficient DSSC is existing^2^,^12^,^13^. For example, plasmonic nanoparticles (Ag, Au) are integrated into photovoltaic devices for light trapping^14^,^15^,^16^. However, use of plasmonic nanoparticles on the photoanode is reported to generate a negative influence named Fano effect due to destructive interference between scattered and unscattered light below the plasmon resonance of nanostructure causing reduced light absorption in DSSC at short wavelength^17^. Application of Aluminum (Al) nanoparticles is reported to overcome such limitation due to Fano effect^17^. Use of porous oxide layer in the photoanodes are reported to be efficient strategy for the layer with higher surface area^18^. Ultrafast electron injection in the DSSC were achieved by doping plasmonic metals^15^,^19^and atomic layer deposition (ALD) of TiO_2_/Al_2_O_3_ after dye adsorption^20^. The use of unidirectional nanotube arrays is shown to exhibit a faster electron diffusion time (τ_d_) along the tube axis^21^. In principle, charge collection efficiency (η_cc_) can be enhanced by reducing τ_d_^22^. However, it has been shown that the nanostructure based photoanodes also exhibit an undesired porous structure and thereby offer poor solar light harvesting^23^. Alternatively, a particular submicron sized TiO_2_ structure called beads was used in whole photoanode or as scattering layer in order to increase charge collection efficiency^9^.

From the brief overview of different synthesis strategies for the optimization of photoanode to account different important parameters for the betterment of the DSSC efficiency, it is most likely that optimization of one parameter may compromise others. Thus finding one-shot optimization strategy of all the parameters for the betterment of solar cell efficiency is “most wanted” and is the motive of the present study. In this work, we have used a particular TiO_2_ structure called carbonate doped mesoporous microstructure (doped MS) in photoanode of a N719 dye containing solar cell. The doped MS prepared using two step non-aqueous solvothermal method in the photoanode essentially satisfies all the key requirements for the enhancement of efficiency of the DSSC. While scattering and electronic band modification increase light trapping, porosity enhances the dye adsorption. The ultrafast electron injection and minimization of electron recombination leading to better solar cell efficiency compared to DSSC with photoanode using undoped MS are also demonstrated.

## Materials and Methods

### Reagents

Titanium isopropoxide, urea, thiourea, TiO_2_ (~21 nm), di-tetrabutylammonium cis-bis(isothiocyanato)bis(2,2′-bipyridyl-4,4′-dicarboxylato)ruthenium(II) (N719), Coumarin 343 (C343), Coumarin 500 (C500), Platinum chloride (H_2_PtCl_6_), lithium iodide(LiI), iodine (I_2_) and 4-tert-butylpyridine (TBP) were purchased from Sigma-Aldrich. Ultrapure water (Millipore System, 18.2 MΩ cm), ethanol and acetone (purchased from Merck) were used as solvents. Analytical-grade chemicals were used for synthesis without further purifications. Fluorine-doped tin oxide (FTO) conducting glass substrates, acquired from Sigma-Aldrich were cleaned by successive sonication with soap water, acetone, ethanol and deionized (DI) water for 15 min, each with adequate drying prior to their use.

### Synthesis of TiO_2_ Microspheres and carbonate doped TiO_2_ Microspheres

The mesoporous TiO_2_ microspheres were synthesized by following the modified previously reported literature^24^,^25^. In brief, 1 mL of titanium isopropoxide was mixed with 15 mL of anhydrous acetone and then stirred for 15 mins. Then the solution was transferred into a 20 mL Teflon lined stainless-steel autoclave and heated at 180 °C for 12 h. The precipitate was collected and washed with acetone and then with ethanol several times. The sample was dried at 60 °C. Carbonate doped TiO_2_ MS were synthesized by mixing the synthesized TiO_2_ MS, urea and thiourea (1 gm, 1.5 gm and 2 gm, respectively). The mixture was ground for 20 mins and then annealed at 400 °C for 5 h. After annealing the powder was washed with water for several times and then dried at 60 °C.

### Sensitization of TiO_2_ MS and doped TiO_2_ MS with C343 and C500 dyes

0.5 mM C343 and C500 solutions were prepared in ethanol under constant stirring for 1 h. The sensitization of TiO_2_ MS and doped TiO_2_ MS with C343 and C500 dyes were carried out at room temperature in the dark by adding TiO_2_ MS and doped TiO_2_ MS into a 0.5 mM dye solution with continuous stirring for 12 h. After the sensitization process, the solution was centrifuged for a few minutes and the supernatant clear solution of unattached dyes was removed. Then the sensitized material was washed with ethanol several times to remove any unattached dye. The nanohybrid was then dried in a water bath and stored in the dark until further use.

### Fabrication of DSSCs

For the fabrication of DSSCs, the counter electrode was made by depositing platinum on the FTO substrates via thermal decomposition of 5 mM platinum chloride (in isopropanol) at 385 °C for 30 min. The N719 coated TiO_2_ MS, doped TiO_2_ MS and TiO_2_ P25 were used as the active electrodes. The photoanodes were fabricated using the following procedure. Initially, TiO_2_ was mixed in water and small amount of acetyl acetonate was added. The mixer was stirred for 5 h. Then triton X-100 was added to the mixer and stirred for few minutes. Finally, the paste was coated on a conducting side of the FTO using the doctor-blade technique. The film was dried at room temperature, sintered at 450 °C for 1 h in a muffle furnace, and then allowed to cool naturally to room temperature. Then the plates were dipped into the ethanolic solution of N719 dye for 18 hour at room temperature. The photoanodes were withdrawn from the solution and immediately rinsed with ethanol. The two electrodes were placed on top of each other with a single layer of 60 μm thick Surlyn (Solaronix) as a spacer between the two electrodes. I^−^/I_3_^−^ was used as electrolyte. The liquid electrolyte composed of 0.5 M lithium iodide (LiI), 0.05 M iodine (I_2_) and 0.5 M 4-tert-butylpyridine (TBP) in acetonitrile was used as the hole conductor and filled in the inter electrode space by using capillary force, through two small holes predrilled on the counter electrode. In all our experiments, the active area of the DSSCs was fixed at 0.49 cm^2^.

### Characterization methods

Field Emission Scanning Electron Microscopy (FESEM, QUANTA FEG 250) was used to investigate the surface morphology of the samples. Transmission electron microscopy (TEM) was carried out using an FEI (Technai S-Twin) instrument with acceleration voltage of 200 kV. The MS samples were taken in ethanol and stirred for few minutes. Then, a drop of sample was placed on a carbon-coated copper grid and dried at room temperature. The particle sizes were determined from micrographs recorded at a high magnification of 100000X. X-ray diffraction (XRD) was used to characterize crystal phase by a PANalytical XPERTPRO diffractometer equipped with Cu Kα radiation (at 40 mA and 40 kV) at a scanning rate of 0.02° S^−1^ in the 2θ range from 20° to 75°. For optical experiments, the steady-state absorption and emission were recorded with a Shimadzu UV-2600 spectrophotometer and a Jobin Yvon Fluoromax-3 fluorimeter, respectively. Picosecond resolution spectroscopic studies were carried out using a commercial time correlated single photon counting (TCSPC) setup from Edinburgh Instruments (instrument response function, IRF = 80 ps, excitation at 375 nm). The details of the experimental setup and methodology were described in our earlier reports^26^,^27^,^28^. The average lifetime (amplitude-weighted) of a multi-exponential decay is expressed as 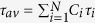. The Förster Resonance Energy Transfer (FRET)^29^,^30^,^31^,^32^has been studied between donor (C500) and acceptor (doped TiO_2_ MS) by following traditional methodology by calculating the Förster distance (R_0_ in Å).

The current density-voltage characteristics of the cells were recorded by a keithley multimeter under irradiance of 100 mW cm^−2^ (AM 1.5 simulated illuminations, Photo Emission Tech). The fill factor (FF) and power conversion efficiency (η) of the solar cells are determined from equations (1) and (2),


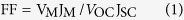



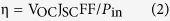


where V_M_ and J_M_ are the voltage and current density at the maximum power output, respectively. J_SC_ and V_OC_ are the short-circuit photocurrent density and open-circuit photovoltage, and P_in_ is the intensity of the incident light (100 mW cm^−2^). The wavelength-dependent photocurrent is measured using a homemade setup with a Bentham monochromator and dual light (tungsten and xenon) sources. Photovoltage decay measurements were carried out after illuminating the cells under 1 Sun. The photovoltage decays after switching off the irradiation were monitored by an oscilloscope (Owon) through computer interface. The decays were fitted with exponential decay functions using origin software.

## Results and Discussion

**Figure 1:**
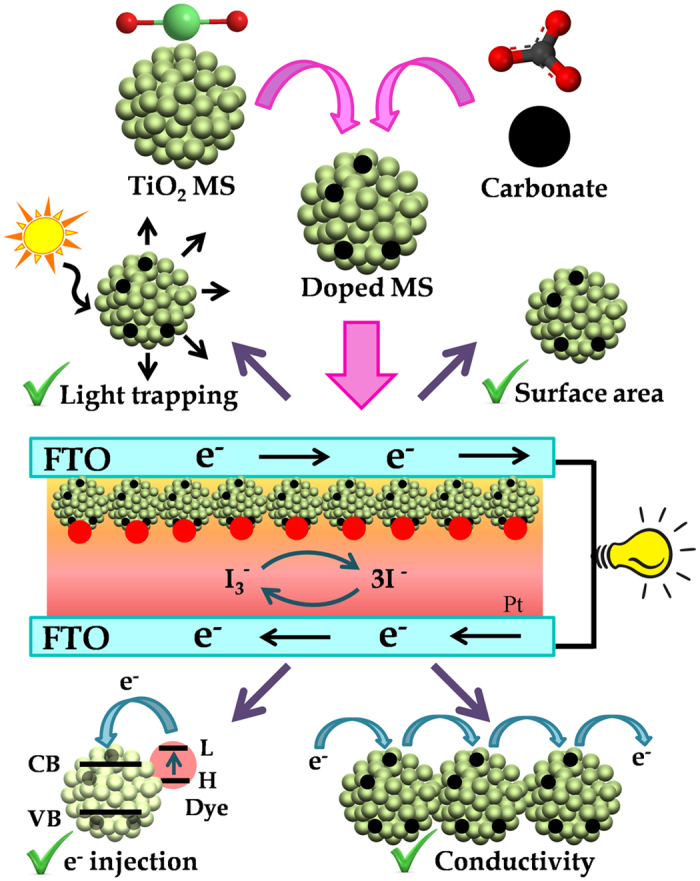
Schematic presentation. Synthesis of doped TiO_2_ MS and their advantages in the dye-sensitized solar cells.

**Figure 2:**
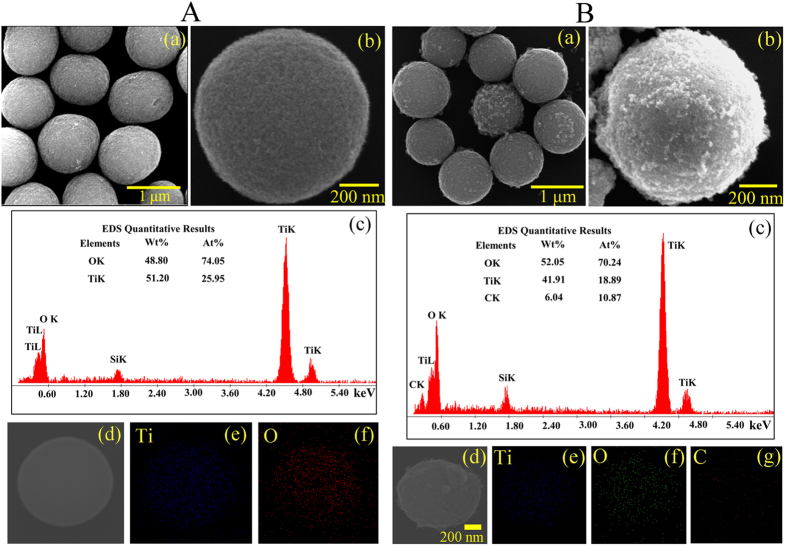
Characterization of TiO_2_ MS and doped TiO_2_ MS. (**A**) SEM images (a,b); EDAX spectrum (c); SEM image of a single particle (d) and Ti, O elemental mapping images of that particle (e,f) of TiO_2_ MS. (**B**) SEM images (a and b); EDAX spectrum (c); SEM image of a single particle (d) and Ti, O, C elemental mapping images of that particle (e–g) of doped TiO_2_ MS.

**Figure 3:**
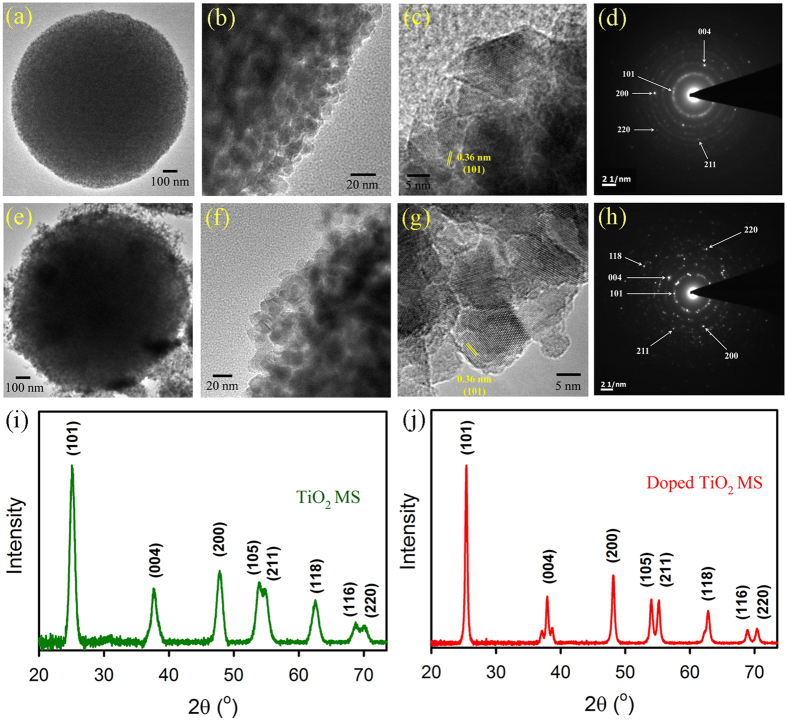
Characterization of TiO_2_ MS and doped TiO_2_ MS. TEM and HRTEM images (**a–c**), SAED patterns (**d**), powder XRD patterns (**i**) of TiO_2_ MS. TEM and HRTEM images (**e–g**), SAED patterns (**h**), powder XRD patterns (**j**) of doped TiO_2_ MS.

The control TiO_2_ MS and doped MS were prepared according to modified procedures reported earlier^24^,^25^,^33^. The positive influences of the doped MS in DSSC performance are schematically shown in Fig. 1. Figure 2-A-a shows scanning electron microscopy (SEM) image of TiO_2_ MS with a diameter range 1–1.1 μm obtained by solvothermal treatment at 180 °C for 12 h. Densely-packed, interconnected TiO_2_ nanocrystals are clearly visible in the high resolution image as shown in Fig. 2-A-b. Figure 2-A-c shows EDAX spectrum of the microspheres with the relative distribution of oxygen and titanium. The elemental EDAX mapping of the microspheres for Ti and O and their distribution in a microsphere are shown in Fig. 2-A-(d-f), consistent with uniform distribution of the element in the sample. No signature of carbon in this sample is evident. In Fig. 2-B-a, we have shown SEM images of the doped MS. The distribution lies in the range of 0.9-1 μm, which is slightly smaller than that of the undoped MS. Similar interconnection of the TiO_2_ nanocrystals with a bit higher porosity is evident from Fig. 2-B-b. The EDAX spectrum (Fig. 2-B-c), the elemental mapping for Ti, O and C (Fig. 2-B-(d-g)) and their atomic percentages clearly show uniform C-doping in the microsphere. In an earlier report on the synthesis of such C-doped nanoparticles, X-ray photoelectron spectroscopy (XPS) was performed and obtained two distinct peaks at 284 eV and 288 eV consistent with C 1s binding energy. While the peak at 284 eV was assigned to carbon adsorbed on the TiO_2_ surface as contaminant, the latter peaking at around 288 eV was concluded to be result of Ti-C bonds^33^. Although high resolution microscopy on the samples was out of the scope of the report, the absorption spectrum of the C-doped samples is consistent with our result (see later). The high resolution transmission electron microscopy (HRTEM) images of the MS before and after doping are shown in Fig. 3. Relatively higher porosity and similar nanoparticle size of 10–20 nm in the case of doped MS compared to undoped counterpart are clear. The additional porosity and surface roughness in the case of doped MS could be result of surface adsorbed carbon as concluded in the earlier XPS study^33^. As the materials were annealed at 450 °C for the preparation of working electrode of the DSSC, we have examined the crystalline condition of the materials after the annealing as shown in Fig. 3(i,j). We have obtained negligibly small difference in the diffractograms after and before annealing. The diffraction peaks of the undoped and doped samples are not only consistent with that reported in the literature^34^, also consistent with the selected area electron diffraction (SAED) patters of the corresponding samples as shown in Fig. 3(d,h).

**Figure 4:**
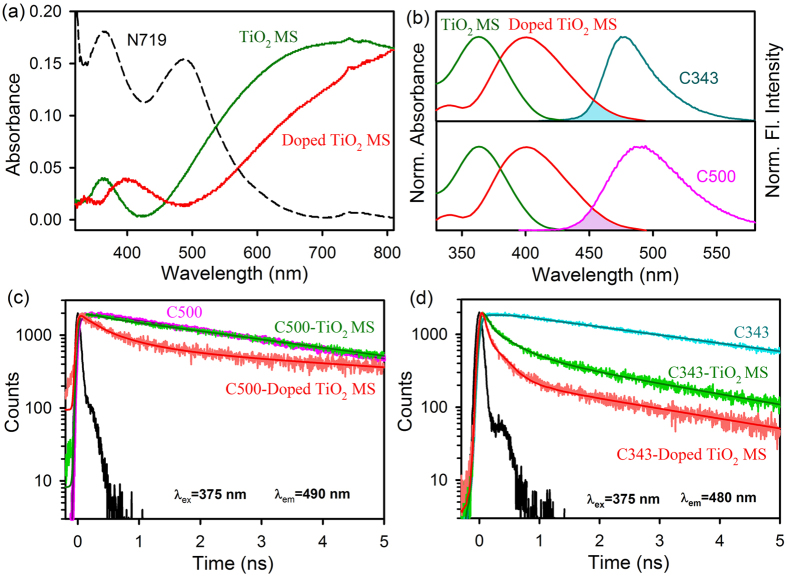
Optical studies of TiO_2_ MS and doped TiO_2_ MS attached to C343 and C500 dyes. (**a**) UV-VIS absorption spectra of TiO_2_ MS and doped TiO_2_ MS. (**b**) Shows the overlap of C343, C500 emission and doped TiO_2_ MS absorption. (**c**) The picosecond-resolved fluorescence transients of C500 (excitation at 375 nm) in the absence (pink) and in the presence of TiO_2_ MS (green) and doped TiO_2_ MS (red) collected at 490 nm. (**d**) The picosecond-resolved fluorescence transients of C343 (excitation at 375 nm) in the absence (cyan) and in the presence of TiO_2_ MS (green) and doped TiO_2_ MS (red) collected at 480 nm.

The absorption spectrum of the doped MS is found to be significantly different from that of undoped counterpart as shown in Fig. 4a. The main electronic band of TiO_2_ at ~360 nm is found to be red shifted to 410 nm upon carbonate doping in addition to significant scattering across the absorption spectrum of N719 in the entire visible solar spectrum. In a recent computational study the density of states (DOS) of undoped and carbonate doped TiO_2_ have been performed^24^. The study showed that the carbonate-doping produced band tail states near the valence and conduction band edges of TiO_2_ decreasing the band gap by about 0.2 eV. The calculation also demonstrated that the band gap in doped TiO_2_ is smaller than that of the undoped one. As shown in Fig. 4b, the red-shift in the absorption spectrum of the doped MS has significant spectral overlap with green light (~500 nm) emission from two coumarin dyes namely C343 and C500 covalently and non-covalently adsorbed at the TiO_2_ surfaces. It has to be noted that peak radiation of solar spectrum is also around 500 nm. Thus ability of absorbing direct sunlight and contributing to the total photoinduced charge carrier in the doped MS are additional advantages over its undoped counterpart. N719 dye does not have emission, thus the coumarin dyes are used to study the electron and energy transfer processes. We have quantified the light absorption in terms of Förster resonance energy transfer (FRET) from the green emitting dye C500 adsorbed to the host surface of the MS. As shown in Fig. 4c, the dye C500 at the doped MS shows faster fluorescence decay compared to C500 at the undoped MS surface revealing resonance energy transfer in the former case^35^. The estimated distance between the dye and the host surface of doped TiO_2_ is found to be 1.53 nm, consistent with the surface adsorption of the dye C500. The ultrafast electron injection of the covalently attached coumarin dye C343 is evident from Fig. 4d. The spectroscopic and fitting parameters are shown in Table 1. It is well known that the covalently adsorbed dye C343 undergoes electron transfer to host TiO_2_ upon photoexcitation^36^. The apparent rate constant, k_nr_, is determined for the nonradiative processes by comparing the lifetimes of C343 in the absence (τ_0_) and the presence (τ) of MS, using the following equation (3)^37^:


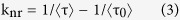


we have estimated that electron transfer rate of the dye C343 at the doped MS (2.8 × 10^9^ s^−1^) is much faster than that at the undoped MS (1.1 × 10^9^ s^−1^). Although a significant spectral overlap of the C343 emission with the absorption band of the doped MS is evident from Fig. 4b, the possibility of FRET in this case can be ruled out for the interference of much faster electron transfer dynamics.

**Figure 5:**
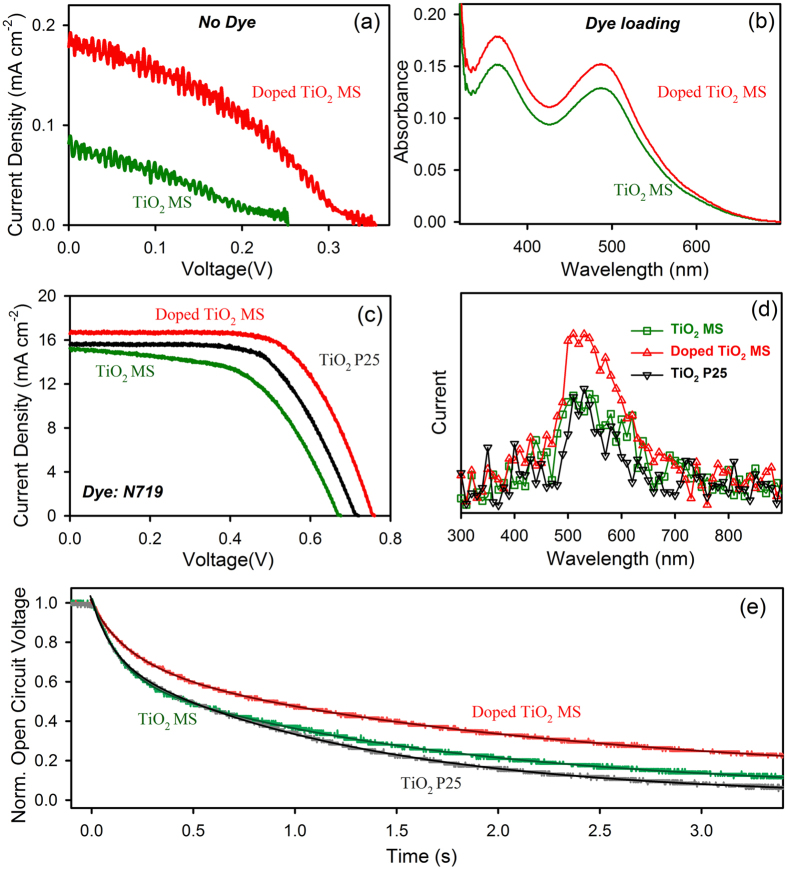
Device performance. I-V characteristics without dye (**a**) and with N719 dye (**c**); (**b**) dye loading; (**d**) wavelength dependent photocurrent response curves and (e) open circuit voltage decay profiles of different DSSCs fabricated with TiO_2_ MS and doped TiO_2_ MS.

The direct light harvesting ability and the better photoconductivity of the doped MS compared to those in its undoped counterpart is evident from I-V characteristics of the solar cell without sensitizing dye (Fig. 5a). Figure 5b shows enhanced dye loading capability of the doped MS because of its better porosity as discussed earlier. The betterment of all the key parameters ultimately enhances the solar cell efficiency as shown in Fig. 5c. The corresponding values of the photovoltaic parameters, such as the short circuit photocurrent density (J_sc_), open circuit voltage (V_oc_), fill factor (FF), power conversion efficiency (η) values are presented in Table 2. The DSSC with doped TiO_2_ MS shows higher energy conversion efficiency of 7.6% compared to that of the undoped MS (5.2%). The enhancement of photocurrent at the peak absorption of the dye N719 is also studied and shown in Fig. 5d. The spectra collected from different cells show good agreement between the wavelength of photocurrent maximum and N719 absorption maximum (λmax = 520 nm). The temporal decay of the open circuit voltage has been monitored for different cells in the dark following a brief period of illumination as shown in Fig. 5e, revealing reduced recombination in the case of DSSC with doped MS. The fitted timescales are presented in Table 3. The open circuit voltage decay reflects the timescales for the recombination processes of the electron at the conduction band of the semiconductor with the oxidized electrolytes^38^. The increase in V_oc_ as evident from Fig. 5c is a consequence of reduction in the back electron transfer^39^.

## Conclusion

Carbonate doped TiO_2_ microspheres were synthesized via two step facile solvothermal route and used as photoanode of a N719 based Dye sensitized solar cell (DSSC). Our single-shot modification in the solar cell design is shown to take care of several key parameters including porosity for dye loading, scattering for light trapping, electron injection for photocurrent and less electron recombination with redox coupling for the enhancement of the efficiency of the solar cell as schematically shown in Fig. 1. We have compared the efficiency of the DSSC with undoped TiO_2_ microsphere and conventional TiO_2_ nanoparticle (P25) and found significant improvement in the light harvesting efficiency upon doping. To our knowledge the realization of carbonate doping solar cells provides a novel pathway to control conversion efficiency in DSSC. Future investigations will focus on tailoring the absorption wavelengths of the sensitizing dye for the light trapping in the NIR region of solar radiation, where 49% power remains un-harvested.

## Additional Information

**How to cite this article**: Seddigi, Z. S. *et al.* Carbonate Doping in TiO_2_ Microsphere: The Key Parameter Influencing Others for Efficient Dye Sensitized Solar Cell. *Sci. Rep.* **6**, 23209; doi: 10.1038/srep23209 (2016).

## Figures and Tables

**Table 1:** Dynamics of picosecond-resolved luminescence transients of C343, C500 and nanohybrids^a^.

Sample	Excitation wavelength (nm)	Detection wavelength (nm)	τ_1_(ns)	τ_2_ (ns)	τ_3_ (ns)	τ_avg_ (ns)
C343	375	480	3.88 (100%)			3.88
C343-TiO_2_ MS	375	480	0.19 (80.7%)	3.04 (19.3%)		0.74
C343-doped TiO_2_ MS	375	480	0.08 (78.3%)	0.35 (16.3%)	3.88 (5.4%)	0.33
C500	375	490	3.25 (100%)			3.25
C500-TiO_2_ MS	375	490	3.25 (100%)			3.25
C500-doped TiO_2_ MS	375	490	0.35 (66.7%)	3.25 (33.3%)		1.31

^a^Numbers in the parenthesis indicate relative weightages.

**Table 2:** Solar cell performance using different active electrodes^b^.

Photoanode	Dye	J_sc_ (mA cm^−2^)	V_oc_ (V)	Fill Factor	η (%)
TiO_2_ MS	No dye	0.08	0.27	38.7	0.01
Doped TiO_2_ MS	No dye	0.18	0.35	38.5	0.02
TiO_2_ MS	N719	15.2	0.68	54.8	5.16
Doped TiO_2_ MS	N719	16.6	0.76	65.6	7.65
TiO_2_ NP P25	N719	15.6	0.72	63.2	6.49

^b^Short-circuit photocurrent densities (J_SC_ cm^−2^), open-circuit voltage (V_OC_) and efficiency (η).

**Table 3:** Dynamics of photovoltage transients of DSSCs fabricated using different active electrodes^c^.

Active Electrode	τ_1_ (S)	τ_2_ (S)	τ_avg_ (S)
TiO_2_ MS	0.13 (38.8%)	1.60 (61.2%)	1.03
Doped TiO_2_ MS	0.21 (33.7%)	2.21 (66.3%)	1.54
TiO_2_ NP P25	0.11 (28.8%)	1.25 (71.2%)	0.92

^c^Numbers in the parenthesis indicate relative weightages.
